# Driver mutations in *TP53* are ubiquitous in high grade serous carcinoma of the ovary

**DOI:** 10.1002/path.2696

**Published:** 2010-05

**Authors:** Ahmed Ashour Ahmed, Dariush Etemadmoghadam, Jillian Temple, Andy G Lynch, Mohamed Riad, Raghwa Sharma, Colin Stewart, Sian Fereday, Carlos Caldas, Anna deFazio, David Bowtell, James D Brenton

**Affiliations:** 1 Functional Genomics of Ovarian Cancer Laboratory, Cancer Research UK Cambridge Research Institute, Li Ka Shing CentreRobinson Way, Cambridge, CB2 0RE, UK; 2 Department of Oncology, University of Cambridge, Hutchison/MRC Research CentreHills Road, Cambridge, CB2 0XZ, UK; 3 Peter MacCallum Cancer CentreMelbourne, Victoria, Australia; 4 Department of Obstetrics and Gynaecology, El-Galaa Maternity Teaching Hospital41 July 26th Street, Cairo 11511, Egypt; 5 Department of Anatomical Pathology, University of Sydney and University of Western Sydney at Westmead HospitalSydney, Australia; 6 Department of Pathology, King Edward Memorial HospitalWestern Australia, Australia; 7 Functional Genomics of Breast Cancer Laboratory, Cancer Research UK Cambridge Research Institute, Li Ka Shing CentreRobinson Way, Cambridge, CB2 0RE, UK; 8 Westmead Institute for Cancer Research, University of Sydney at Westmead Millennium Institute, Westmead HospitalNew South Wales, Australia; 9 Department of Biochemistry and Molecular Biology, University of MelbourneParkville, Victoria, Australia

**Keywords:** p53, high-grade pelvic serous carcinoma, ovarian cancer, DNA sequence analysis, array-based genomic hybridization, histopathology, clinical outcome, BRCA

## Abstract

Numerous studies have tested the association between *TP53* mutations in ovarian cancer and prognosis but these have been consistently confounded by limitations in study design, methodology, and/or heterogeneity in the sample cohort. High-grade serous (HGS) carcinoma is the most clinically important histological subtype of ovarian cancer. As these tumours may arise from the ovary, Fallopian tube or peritoneum, they are collectively referred to as high-grade pelvic serous carcinoma (HGPSC). To identify the true prevalence of *TP53* mutations in HGPSC, we sequenced exons 2–11 and intron–exon boundaries in tumour DNA from 145 patients. HGPSC cases were defined as having histological grade 2 or 3 and FIGO stage III or IV. Surprisingly, pathogenic *TP53* mutations were identified in 96.7% (*n* = 119/123) of HGPSC cases. Molecular and pathological review of mutation-negative cases showed evidence of p53 dysfunction associated with copy number gain of *MDM2* or *MDM4*, or indicated the exclusion of samples as being low-grade serous tumours or carcinoma of uncertain primary site. Overall, p53 dysfunction rate approached 100% of confirmed HGPSCs. No association between *TP53* mutation and progression-free or overall survival was found. From this first comprehensive mapping of *TP53* mutation rate in a homogeneous group of HGPSC patients, we conclude that mutant *TP53* is a driver mutation in the pathogenesis of HGPSC cancers. Because *TP53* mutation is almost invariably present in HGPSC, it is not of substantial prognostic or predictive significance. Copyright © 2010 Pathological Society of Great Britain and Ireland. Published by John Wiley & Sons, Ltd.

## Introduction

The gene *TP53* that encodes the tumour suppressor protein p53 is amongst the most commonly mutated genes in human cancer [1]. The frequent presence of *TP53* mutations in ovarian cancer has been suggested for almost two decades [2,3] and during this time, approximately 70 publications have described the relationship between *TP53* mutation status and clinical outcome [4]. For such an intensively studied question, there is a surprising degree of inconsistency in the published data for both the prevalence of mutation and association with prognosis or chemotherapy response.

A recent analysis of 64 publications reporting the relationship of *TP53* mutation to clinical outcome found that only six studies fulfilled minimum quality criteria for the method of detecting mutations or assignment of clinical response [5]. One of the most consistent limitations of previous p53 studies is a reliance on p53 immunostaining as a surrogate marker for *TP53* mutation [4], even though this leads to an unacceptable number of misclassifications [6]. Sequencing of tumour DNA is the gold standard to detect different types of mutation and relate these to clinical outcome. However, a recent review of 12 studies that sequenced *TP53* in ovarian cancer found variable mutation frequencies of 15–80% [5]. Inconsistencies between these studies may relate to whether the whole gene was or only the most commonly mutated exons were sequenced. Additionally, most studies were confounded by the inclusion of cases with different histological types, stages, and grades of tumours [4].

Determining the true prevalence of *TP53* mutation is critical for understanding the pathogenesis of high-grade serous cancers arising as ovarian, Fallopian tube or primary peritoneal cancer. Although previously assumed to arise from the ovary, pathological examination of the Fallopian tube from carriers with *BRCA1* or *BRCA2* mutation has demonstrated that tubal intraepithelial carcinomas (TICs) arising from secretory epithelial cells in the fimbria are the early invasive lesion in high-grade serous (HGS) carcinoma [7,8]. In addition, TICs are found in patients without evidence of germline *BRCA1* or *BRCA2* lesions and show concomitant *TP53* mutations in the tube and in pelvic metastases, further supporting the model that TIC represents the origin of HGS carcinoma. HGS cancers may also arise from the peritoneum and although these may have different epidemiological risk factor profiles [9], they exist in a clinical continuum with ovarian and Fallopian tube cancers. Hence we use the term high-grade pelvic serous carcinoma (HGPSC) [10] to recognize the frequent lack of clarity regarding primary site in ovarian/Fallopian tube/peritoneal cancers and their overall biological and clinical similarity.

To address the limitations of previous studies and determine accurately the relationship between *TP53* mutation and HGPSC, we designed pilot and validation studies to measure the mutation frequency in a homogeneous group of advanced stage HGPSC cancers from a large population-based cohort.

## Materials and methods

### Samples and study design

The results of this study are presented in accordance with reporting recommendations for tumour marker prognostic studies (REMARK) criteria [11]. Supplementary Figure 1 (Supporting information) summarizes the flow of patients through the pilot and validation study, including the number of patients included in each stage of the analysis and reasons for exclusion. Patients (*n* = 123) were selected from the Australian Ovarian Cancer Study (AOCS), a population-based, multicentre translational study that comprised prospective collection of bio-specimens and clinical and epidemiological data from patients with primary epithelial ovarian, primary peritoneal, and Fallopian tube cancer diagnosed between 2001 and 2005. Patients treated at Westmead Hospital, Sydney between 1992 and 2002 (*n* = 22) were also included in the pilot cohort, making an overall sample size of 145 HGPSCs. All women in the pilot cohort received first-line platinum-based chemotherapy (cisplatin or carboplatin) with (*n* = 39) or without (*n* = 6) the taxane paclitaxel. Similarly, the majority of patients in the validation set received combination platinum–taxane treatment (*n* = 93) or platinum monotherapy (*n* = 4). Three patients (*n* = 3) did not receive any chemotherapy, including one patient with stage I cancer who declined treatment and two patients who were too ill to receive chemotherapy. All patients were prospectively consented using a protocol approved by the human research ethics committees at multiple participating clinical and research centres. Further details of the AOCS cohort can be found at http://www.aocstudy.org. Statistical design and power calculations are described in the Supporting information, Supplementary methods.

### *TP53* sequencing

Coding *TP53* exons 2–11 were amplified using primers that encompassed the entire exon and exon–intron boundaries. Purified PCR products were se- quenced using an ABI 3100 genetic analyser (Applied Biosystems, Warrington, UK). Sequencing reactions were performed in forward and reverse directions. Mutational analysis was performed using SeqScape® Software v2.6 (Applied Biosystems). Sequence traces were aligned against the *TP53* reference sequence NC_000017. Independent, blinded sequencing of cases from the pilot study was performed by the CR-UK Mutation Detection Facility, Clinical Sciences Building, Level 6, St James's University Hospital, Leeds, UK.

### Characterization of mutation-negative HGPSC cancers

For single nucleotide polymorphism (SNP) mapping assay, SNP microarray data analysis, quantitative PCR (qPCR), and immunohistochemistry, the reader is referred to the Supporting information, Supplementary methods.

### aCGH data

Raw data files from aCGH experiments are available from the Gene Expression Omnibus http://www.ncbi.nlm.nih.gov/geo/ (Accession number GSE19416).

## Results

### Both treatment-responsive and primary resistant HGPSCs have a high frequency of *TP53* mutation

To estimate the number of samples required to detect a significant association between *TP53* mutations and survival, we performed a pilot study with 45 HGPSCs (ovarian, Fallopian tube, and primary peritoneal). The cohort included 20 chemotherapy-resistant patients who had tumour progression within 6 months of completion of treatment and 25 responsive patients who did not progress for at least 9 months. The two groups showed no statistically significant differences in age, tumour stage, residual disease or type of chemotherapy (Table 1). Review of pathology reports showed one patient with low-grade serous (LGS) carcinoma (case 450) and this case was excluded from further analysis. Unexpectedly, pathogenic *TP53* mutations were identified in 43 out of 44 patients included in the study (Supporting information, Supplementary Table 1 and Supplementary Figure 1). Mutations were compared to Release 13 of the IARC *TP53* mutation database [12] and 20 cases had rare *TP53* mutations, defined as less than ten occurrences (Supporting information, Supplementary Table 1). From these, 12 were randomly selected and independently confirmed by a clinical reference laboratory blinded to our results (data not shown). Further analysis of mutations is described below.

**Table 1:** A comparison between the clinical characteristics of the chemotherapy-resistant and -responsive patients in the pilot study

	Resistant (*n* = 20)	Responsive (*n* = 25)	*p* (test)
PFS (months)			
Median	10.2	23.3	< 0.001 (log rank)
OS (months)			
Median	21.4	57.3	< 0.001 (log rank)
Age (years)			
Median	58.26	58.7	0.7 (Wilcoxon rank sum)
Range	22.9–78	38.1–77.7	
Stage (*n*)			
III	20	23	0.5 (Fisher)
IV	—	2	
Chemotherapy (*n*)			
Adjuvant	19	25	0.4 (Fisher)
Neo-adjuvant	1	0	
Residual disease (*n*)			
No residual disease	2	5	0.2 (Fisher)
≤1 cm	11	8	
> 1 cm and ≤2 cm	5	5	
> 2 cm	1	6	
Unknown	1	1	
TP53 (*n*)			
Wild type	1	0	0.4 (Fisher)
Mutant	19	25	

OS = overall survival; PFS = progression-free survival, defined as the period from diagnosis to the date of documented progression. Six samples (2 resistant, 4 responsive) did not have precise values for size < 2 cm.

### Confirmation of high frequency of *TP53* mutation in an unselected population-based cohort of serous cancers

To exclude the possibility that the high rate of *TP53* mutations was biased by the selection criteria used in the pilot study, we obtained additional serous cancer cases that were a representative subset of a large population-based series (Table 2). Mutations were detected in 76/82 advanced-stage HGPSCs from these cases, with no significant difference between the percentage of mutations in the matched validation study compared with the pilot study (*p* = 0.3; Fisher exact test).

**Table 2:** Clinical characteristics of the validation study patients

	Validation set (*n* = 82
PFS (months)	
Median	13.3
OS (months)	
Median	34
Age (years)	
Median	61.3
Range	40.4–79.3
Stage (*n*)	
III	73
IV	9
Grade (*n*)	
2	18
3	63
Unknown	1
Chemotherapy (*n*)	
Adjuvant	71
Neo-adjuvant	11
Residual disease (*n*)	
No residual disease	16
≤1 cm	26
> 1 cm and ≤2 cm	5
> 2 cm	25
Unknown	10
TP53 (*n*)	
Wild type	6
Mutant	76

OS = overall survival; PFS = progression-free survival.

### *TP53* mutations arise at high frequency in exons 2–4 and 9–11 in HGPSC carcinoma

We analysed the 119 mutations found in 126 HGPSC cases from the pilot and validation set (Supporting information, Supplementary Table 1). As expected, the majority of *TP53* mutations were missense mutations (56.3%, *n* = 67/119) occurring in exons 4–8 (Supporting information, Supplementary Table 1). The most frequently mutated codon was 273 (*n* = 9/67 cases, 13.4%) comprising R273C, R273H, and R273L mutants. Analysis of exon and splice sites for exons 2–4 and 9–11, outside the DNA binding domain, showed 12 (10.1%) and 10 (8.4%) mutations, respectively, which taken together comprised 18.5% (*n* = 22/119) of all mutations in our series. Of note, a total of eight mutations affected exon 10 (6.7%) in the tetramerization domain (codons 324–356) and all were predicted to cause truncation of the protein. Four of these mutations occurred at g.16915 (codon 342). In 717 serous sub-type ovarian, Fallopian, and primary peritoneal cancer cases in Release 13 of the IARC *TP53* database, 30 (4.2%) had mutations in exons 2–4, 18 had mutations affecting exon 10 alone (2.5%), and 20 (2.8%) had mutations in exons 9–11. Twenty-two mutations from our series (*n* = 22/119, 18.5%) were not present in the IARC database. With the exception of one, all were insertions or deletions (Supporting information, Supplementary Table 1). The remaining novel mutation in case 434 was at position g.11602_11603GC > AA, creating a unique tandem nonsense mutation.

### Low-stage HGPSC cases have a high frequency of *TP53* mutations

The very high prevalence of mutation in high-stage cases suggested that *TP53* was an early event in the pathogenesis of HGPSC. From the validation set, 15 cases were stage 1 or 2 HGPSC and 13/15 (86.7%) had *TP53* mutation (Supporting information, Supplementary Table 1). The negative cases 41307 and 60111 had mixed endometrioid/HGS or grade 1 pathology, respectively. As for high-stage cases, the majority of mutations (*n* = 10/13; 76.9%) were missense mutations in exons 5–8. There was one nonsense mutation in exon 10 (7.7%) and two splice junction mutations (15.4%).

### Mutation-negative cases have alternative mechanisms of p53 dysfunction

**Figure 1:**
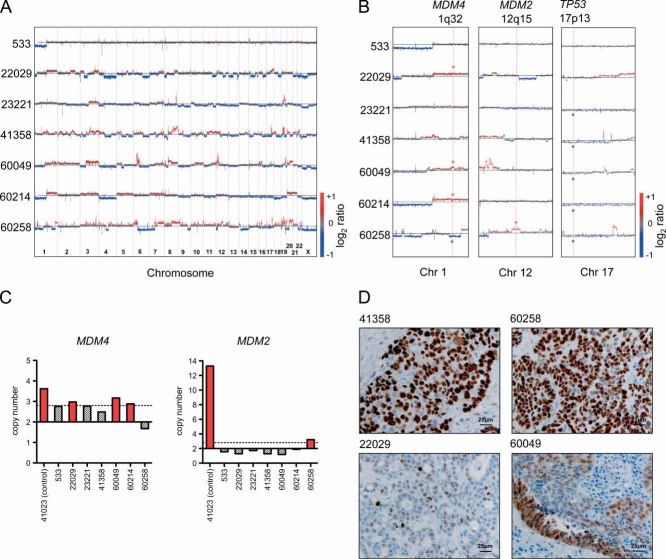
Characterization of *TP53* mutation-negative cancer samples. (A) Summary plots of whole genome DNA copy number data for seven mutation-negative samples. Red indicates chromosomal gain and blue shows regions of chromosomal loss. (B) High-resolution DNA copy number analysis of *MDM2, MDM4*, and *TP53* loci in each sample. The dotted line indicates the position of the gene within the region. Samples with chromosomal gain or loss are marked with a red or blue asterisk, respectively. (C) Confirmation of *MDM2* and *MDM4* copy number gain using quantitative PCR of tumour DNA. Samples with gain are indicated in red. (D) Immunohistochemical staining of p53 protein in the following selected mutation-negative cases: case 41358; case 60258 (*MDM2* gain); case 22029 (*MDM4* gain); and case 60049 (*MDM4* gain).

We considered the possibility that *TP53* mutations in the seven mutation-negative samples (pilot *n* = 1, validation *n* = 6) were missed because of contamination with normal DNA. Although all samples with significant stroma were needle-dissected prior to sequencing, pathological review of the mutation-negative cases indicated that they did not have increased stroma compared with mutation-positive cases (data not shown). Additionally, array-based comparative genomic hybridization (aCGH) showed that all mutation-negative cases had DNA copy number abnormalities (DCNAs), and the least aberrant, case 533, had distinct loss of chromosome arm 1p (Figure 1A). No samples showed evidence of homozygous deletion of the *TP53* locus (Figure 1B).

Pathology review of the mutation-negative cases resulted in the re-classification of case 60214 as LGS carcinoma, case 23221 as high-grade carcinoma of uncertain primary site, and case 533 as mixed LGS/HGS type (Supporting information, Supplementary Table 2). Furthermore, aCGH analysis of cases 533 and 60214 did not show high-frequency DCNAs that are typical of HGPSC [11]. The original diagnosis of HGPSC was confirmed in the remaining four cases (22029, 41358, 60049, and 60258). To further exclude the possibility of pathological misclassification of endometrioid, clear cell or LGS tumours in the mutation-negative cases, we also tested for mutations commonly associated with these subtypes in *KRAS* (exon 2), *BRAF* (exon 15), *CTNNB1* (exon 3), and *PIK3CA* (exons 10 and 21). Case 60214 had a missense mutation (G12C) in exon 2 of *KRAS*, consistent with LGS origin. No other mutations were detected (data not shown).

Other mutation-independent mechanisms of inactivation of the p53 pathway were investigated. p53 is targeted for degradation through the activity of MDM2 protein and is also regulated by the related MDM4 protein [13,14]. Amplification of *MDM2* is associated with loss of p53 activity in some solid tumours [15,16]. aCGH analysis of the remaining four cases showed copy number gain of *MDM4* in cases 22029 and 60049, and of *MDM2* in case 60258 (Figure 1B). These observations were confirmed by qPCR (Figure 1C). Interestingly, both cases with *MDM4* gain showed heterogeneous p53 immunostaining, including cytoplasmic staining (Figure 1D). Case 60258 with *MDM2* gain showed strong nuclear staining for p53 protein (Figure 1D). Interestingly, the remaining case with no MDM2 or MDM4 gain (41358) showed strong nuclear p53 immunostaining (Figure 1D).

In summary, the overall *TP53* mutation rate for selected HGPSC cases in the pilot and validation cohorts was 94.4% (*n* = 119/126). After removing the three mutation-negative cases with atypical or LGS histology, the adjusted overall rate was 96.7% (*n* = 119/123). In the remaining four mutation-negative HGPSC cases, three showed evidence of p53 dysfunction with increased p53 nuclear staining or cytoplasmic mislocalization that was correlated with gains at the *MDM2/4* loci.

### *TP53* mutation type is not prognostic in HGPSC

It has been previously reported that the type of *TP53* mutation may predict outcome in ovarian cancer [17]. Although our study was not adequately powered to detect small effects, we tested for trends in the data. For this analysis, we used all presumed HGPSC cases from the pilot study and the validation cohort (*n* = 127, *n* = 120 had *TP53* mutation; Tables 3 and 4). There was no indication of significant association between the frequency of *TP53* mutation or the type of mutation (missense versus not) and progression-free survival or overall survival. Similarly, whether or not the missense mutation affected the DNA binding motifs formed by the L2 and L3 loops (codons 164–194 and 237–250, respectively) or the LSH motifs (codons 119–135 and 272–287) [18] had no significant effect on progression-free survival or overall survival (Tables 3 and 4). There was also no effect of codon 72 polymorphism (Arg, Pro or heterozygous) on prognosis in any of the HGPSCs with mutations (data not shown) or in tumours with missense mutations (Tables 3 and 4). There was no significant interaction between the type of missense mutation (DNA binding or not) and codon 72 polymorphism (data not shown).

**Table 3:** The type of *TP53* mutation does not influence progression-free survival

Index category (*n*)	Comparison (*n*)	Hazard ratio	CI	Log-rank *p* value
Mutant (120)	Wild type (7)	1.4	0.6–3.1	0.5
Not missense (52)	Missense (68)	0.9	0.6–1.4	0.8
DBM (44)	NDBM (24)	1	0.6–1.7	1

DBM = DNA binding mutations; NDBM = non-DNA binding mutations.

**Table 4:** The type of *TP53* mutation does not influence overall survival

Index category (*n*)	Comparison (*n*)	Hazard ratio	CI	Log-rank *p* value
Mutant (120)	Wild type (7)	1.7	0.6–4.6	0.3
Not missense (52)	Missense (68)	1	0.7–1.6	1
DBM (44)	NDBM (24)	1.3	0.7–2.4	0.4

DBM = DNA binding mutations; NDBM = non-DNA binding mutations.

## Discussion

By addressing the limitations of numerous other studies of *TP53* mutation in ovarian cancer, we have demonstrated for the first time that HGPSCs have the highest frequency of p53 mutation of any solid cancer. Approximately two-thirds of mutations occurred in exons 5–8, known to be mutation hotspots for *TP53*. By sequencing exons 2–4 and 9–11, we identified 22 mutations (*n* = 22/126, 17.5%) that would not have otherwise been identified in our series and are poorly represented in previous reports. The previously unreported mutations that we have discovered are mostly insertions/deletions predicted to result in truncated proteins, but it is notable that HGPSC cases have frequent involvement of exon 10 and specifically involvement of position g.16915 (codon 342) in the oligomerization domain of p53. This region also contains a putative nuclear export signal for p53 and a recent study of a novel missense mutation at codon 351 has shown that K351N results in significant loss of p53 tetramerization, reduced BAX activation, and reduced nuclear export of p53 [19]. Cytosolic export of p53 is required for cisplatin-induced apoptosis in A2780 cells, suggesting that combined loss of tetramerization and nuclear export is critical for platinum resistance.

Of the mutation-negative cases, approximately half were reclassified on pathological review as unrepresentative of HGPSC, including two tumours that were LGS and associated with borderline tumour. Serous cancers may follow two distinct routes to malignancy [20]. A minority of so-called type I tumours progress from borderline tumours, whereas the majority of HGS (type II) tumours arise with no evidence of LGS or borderline tumours. Borderline serous tumours and their type I invasive counterparts are generally low grade, have relatively limited genomic DNA copy number change, have frequent activating RAS pathway mutations, and have substantially lower rates of *TP53* mutation compared with type II tumours [20–23]. Detailed analysis of six cases of HGPSC arising from LGS and borderline tumours showed no *TP53* mutation [24]. Consistent with this model, mutation-negative case 533 had both LGS and HGS components and a bland aCGH profile.

Exclusion of LGS cases and mixed high-grade serous cases increased the *TP53* mutation frequency to approximately 97% of HGPSCs. Of the remaining mutation-negative cases, three may have had inactivation of the p53 pathway through chromosomal gain at the *MDM2* or *MDM4* loci or potentially other genes that alter p53 function in *trans* such as *RFWD2* and *RCHY1* [25,26].

We are aware of over 70 publications that have sought to relate *TP53* mutation with clinical outcome in ovarian cancer, with conflicting conclusions about the importance of *TP53* mutations for tumour aggressiveness, response to treatment, and survival. There is no sign that a conclusion is at hand: since 2008, there have been a number of papers relating *TP53* mutation and clinical outcome [27–30], with evidence presented for [29] and against [28] mutation being a significant prognostic factor. A recent meta-analysis of 62 studies concluded that *TP53* mutation had a modest impact on survival in ovarian cancer but the effect was insufficient to support clinical application [4]. Whilst meta-analyses such as that of de Graeff *et al* [4] have the potential to detect subtle trends that may be missed in all but the largest individual studies, they are confounded if there is a substantial false-negative rate, as our findings indicate. Given the near ubiquitous occurrence of *TP53* mutation, it difficult to conclude that it can be of significant prognostic or predictive significance in HGPSC. We have not, however, excluded the possibility that specific mutations can influence prognosis or response to therapy, an important consideration given the mounting evidence that some *TP53* mutations have specific neomorphic functions [1]. It is thought that inactivation of the p53 pathway is a common, perhaps mandatory, event in all solid cancers [31]. What is particularly unusual about HGPSC is an almost total reliance on mutation *TP53* itself for pathway inactivation, perhaps reflecting the requirement of certain tissues for neomorphic mutations for their transformation [1]. Future work will need to address interactions between specific mutations and other loci such as *PTEN* and *CDKN2A* [32,33].

Our findings have important implications for the new understanding of the pathogenesis of HGPSC. p53 immunostaining and mutation appear to be a feature of early-stage Fallopian tube lesions from *BRCA1/2* mutation carriers [7], suggesting that p53 dysfunction is essential for early tumourigenesis of HGPSC. The notion that *TP53* mutation is required to allow survival of *BRCA*-deficient ovarian precursor lesions is consistent with the extremely high rates of *TP53* mutation in breast cancers arising in *BRCA1* mutant women [34,35]. BRCA dysfunction may also occur in sporadic HGPSC via a variety of inactivating mechanisms but the very high rate of *TP53* mutation in our series of sporadic HGPSC cases suggests that most are ‘BRCA-like’ with deficiencies for *BRCA* or closely related DNA repair pathways [36].

It has become increasingly clear that ovarian cancer is a series of distinctly different diseases with different aetiologies [23,37] and that the development of reliable biomarkers must be subtype-specific [38]. For example, several biomarkers that appeared to be prognostic in a cohort of all major subtypes of ovarian cancer were not informative within homogeneous subtypes. Our findings provide another example of the importance of refining the analysis of ovarian biomarkers by focusing on the most clinically significant group, HGPSC.

The key oncogenic and tumour suppressor genes for HGPSC have not been identified and as it has high rates of genomic instability, many of the alterations described may be passenger mutations. Our results for the prevalence of *TP53* mutation may be conservative as our prospectively determined selection criteria excluded low-stage HGPSC cases (*n* = 15). This was because true low-stage HGPSC is a rare entity and often suggests misdiagnosis of LGS histiotype. However, 13/15 of these cases had *TP53* mutation. Our data are therefore consistent with mutant *TP53* being an essential driver mutation early in the pathogenesis of HGPSC. Future studies will need to focus on studying the biological significance of the different types of *TP53* mutation in HGPSC and developing p53 synthetic-lethal therapies for patients with this disease.

## SUPPORTING INFORMATION ON THE INTERNET

The following supporting information may be found in the online version of this article.

**Supplementary experimental procedures.**

**Table S1.** Samples listed by AOCS number with results of somatic *TP53* sequencing.

**Table S2.** Summary of clinical, pathology and molecular review of mutation-negative serous carcinomas.

**Figure S1.** Summary of all cases included in the pilot and validation sets.
