# Supplementary material for: Driver mutations in TP53 are ubiquitous in high grade serous carcinoma of the ovary
Source: J Pathol. 2010 May;221(1):49–56. doi: 10.1002/path.2696 (PMC3262968; doi:10.1002/path.2696)
Supplement: Supplementary file 1 [file path0221-0049-SD1.pdf]

Supplementary Table 1. Samples listed by AOCS number with results of somatic *TP53* sequencing. Annotations for columns 3–10 are as used in the IARC *TP53* database R13 (see `http://www-p53.iarc.fr/Help.html`) and genomic nucleotide positions for mutations use Genbank NC_000017 as the reference sequence. The frequency of specific *TP53* mutations in the IARC *TP53* R13 and the codon 72 polymorphism are also shown. Twelve samples from the pilot set were independently sequenced by an external reference laboratory (indicated by ⋆). Fifteen serous carcinoma samples from the validation set were stage I or II (indicated by †) and three were low-grade serous carcinomas (indicated by ‡).

Sample	Genomic variant	Coding nt	ExonIntron	Prot Description	Codon no	Description	Mutant codon	Mutant AA	Effect	IARC freq.	Codon 72 status
Pilot Study											
152⋆	g.12466_12467insGCGCCA	c.478	5-exon		160	ins6			other	0	Arg
153⋆	g.11211_11212insCGTT	c.89	3-exon		30	ins4			FS	0	Arg
374	g.12705delT	c.636	6-exon		212	del1			FS	0	Arg
379	g.12643c>T	c.574	6-exon	p.Q192X	192	C>T	TAG	Stop	nonsense	84	Pro
381⋆	g.11437delc	c.206	4-exon		69	del1			FS	1	Pro
424	g.13798g>a	c.818	8-exon	p.R273H	273	G>A	CAT	His	missense	733	Arg
434⋆	g.11602_11603GC>AA	c.371	4-exon	p.C124X	124	GC>AA	TAA	Stop	nonsense	0	Arg
450⋆	g.12475t>g	c.487	5-exon	p.Y163D	163	T>G	GAC	Asp	missense	4	Arg
474	g.12491a>c	c.503	5-exon	p.H168P	168	A>C	CCC	Pro	missense	12	Pro
484⋆	g.12431_12432delAT	c.443	5-exon		148	del2			FS	1	Arg
488	g.13371g>a	c.734	7-exon	p.G245D	245	G>A	GAC	Asp	missense	147	Arg
493⋆	g.13751_13765delTATCCTGAGTAGTGG	c.783 − 12	7-intron		0 (261)	del15			other	0	Arg
496	g.12512g>A	c.524	5-exon	p.R175H	175	G>A	CAC	His	missense	1063	Arg
500	g.12475t>c	c.487	5-exon	p.Y163H	163	T>C	CAC	His	missense	21	Arg
502	g.13804g>a	c.824	8-exon	p.C275Y	275	G>A	TAT	Tyr	missense	70	Pro
518	g.13380g>a	c.743	7-exon	p.R248Q	248	G>A	CAG	Gln	missense	799	Arg
525	g.13797c>T	c.817	8-exon	p.R273C	273	C>T	TGT	Cys	missense	611	Arg
533											Arg
541	g.13350g>a	c.713	7-exon	p.C238Y	238	G>A	TAT	Tyr	missense	79	Arg
546	g.16936g>t	c.1045	10-exon	p.E349X	349	G>T	TAA	Stop	nonsense	6	Arg
552	g.13371g>t	c.734	7-exon	p.G245V	245	G>T	GTC	Val	missense	71	Arg
575	g.12647a>g	c.578	6-exon	p.H193R	193	A>G	CGT	Arg	missense	82	Arg
22023	g.12481c>t	c.493	5-exon	p.Q165X	165	C>T	TAG	Stop	nonsense	41	Arg
22037⋆	g.13368delG	c.731	7-exon		244	del1			FS	3	Arg
23026	g.12627a>C	c.560 − 2	5-intron		0	A>C			splice	4	Arg
23030	g.13407t>c	c.770	7-exon	p.L257P	257	T>C	CCG	Pro	missense	12	Arg
23053⋆	g.12381delC	c.393	5-exon		131	del1			FS	0	Arg
32048	g.12363g>T	c.376 − 1	4-intron		0	G>T			splice	5	Pro
34019	g.12512g>a	c.524	5-exon	p.R175H	175	G>A	CAC	His	missense	1063	Arg
34059	g.13798g>a	c.818	8-exon	p.R273H	273	G>A	CAT	His	missense	733	Pro
41079	g.12377t>a	c.389	5-exon	p.L130H	130	T>A	CAC	His	missense	2	Arg
44186	g.13851a>t	c.871	8-exon	p.K291X	291	A>T	TAC	Stop	nonsense	5	Arg
44189⋆	g.13401_13426delTCACACTGGAAGACTCCAGGTCAGGinsA	c.764	7-exon		255	del25ins1			FS	0	Arg/Pro
44242	g.12487c>t	c.499	5-exon	p.Q167X	167	C>T	TAG	Stop	nonsense	33	Arg
44306	g.13349t>G	c.712	7-exon	p.C238G	238	T>G	GGT	Gly	missense	10	Arg
44313	g.11553_11558delGGTTTC	c.322	4-exon	p.G108_F109del	108	del6			other	2	Arg
51005	g.13409g>a	c.772	7-exon	p.E258K	258	G>A	AAA	Lys	missense	60	Arg
51016⋆	g.11573_11574insG	c.342	4-exon		114	ins1			FS	0	Arg/Pro
51080	g.12706c>t	c.637	6-exon	p.R213X	213	C>T	TGA	Stop	nonsense	259	Arg
60139	g.13320_13323delACTG	c.683	7-exon		228	del4			FS	0	Arg
70047	g.12476a>G	c.488	5-exon	p.Y163C	163	A>G	TGC	Cys	missense	137	Arg

70048	g.13359c>t	c.722	7-exon	p.S241F	241	C>T	TTC	Phe	missense	94	Arg
70049⋆	g.11499_11500delTC	c.268	4-exon		90	del2			FS	0	Arg
70054	g.13798g>a	c.818	8-exon	p.R273H	273	G>A	CAT	His	missense	733	Arg
70094	g.12524a>g	c.536	5-exon	p.H179R	179	A>G	CGT	Arg	missense	139	Arg

Validation Study

20019†	g.12521a>c	c.533	5-exon	p.H178P	178	A>C	CCC	Pro	missense	10	Arg
20027	g.12440c>a	c.452	5-exon	p.P151H	151	C>A	CAC	His	missense	33	Pro
20032	g.12476a>g	c.488	5-exon	p.Y163C	163	A>G	TGC	Cys	missense	137	Arg
20041†	g.12548g>a	c.599 + 1	5-intron		0	G>A			splice	25	Pro
20074	g.13382a>g	c.745	7-exon	p.R249G	249	A>G	GGG	Gly	missense	43	Arg
22013	g.13896c>t	c.916	8-exon	p.R306X	306	C>T	TGA	Stop	nonsense	136	Arg
22020	g.12365a>g	c.377	5-exon	p.Y126C	126	A>G	TGC	Cys	missense	14	Arg
22029											Pro
22046	g.13867_13868delACinsT	c.887	8-exon		296	del2ins1			FS	0	Arg
22047	g.13818a>g	c.838	8-exon	p.R280G	280	A>G	GGA	Gly	missense	39	Pro
22048†	g.13900g>c	c.919 + 1	8-intron		0	G>C			splice	6	Arg
22057	g.12653t>c	c.584	6-exon	p.I195T	195	T>C	ACC	Thr	missense	84	Arg
22058	g.13384delG	c.747	7-exon		249	del1			FS	8	Arg
23055	g.12411c>a	c.423	5-exon	p.C141X	141	C>A	TGA	Stop	nonsense	19	Arg
23070	g.12410g>a	c.422	5-exon	p.C141Y	141	G>A	TAC	Tyr	missense	93	Arg
23072	g.16930delG	c.1039	10-exon		347	del1			FS	0	Pro
23074†	g.12728a>g	c.659	6-exon	p.Y220C	220	A>G	TGT	Cys	missense	312	Pro
23077	g.12525t>a	c.537	5-exon	p.H179Q	179	T>A	CAA	Gln	missense	8	Arg
23098†	g.12439c>t	c.451	5-exon	p.P151S	151	C>T	TCC	Ser	missense	88	Arg
23106	g.13359c>a	c.722	7-exon	p.S241Y	241	C>A	TAC	Tyr	missense	17	Arg
23116	g.12526_12528delGAGinsA	c.538	5-exon		180	del3ins1			FS	0	Arg
23128	g.13836g>a	c.856	8-exon	p.E286K	286	G>A	AAA	Lys	missense	78	Arg/Pro
23143	g.13867delA	c.887	8-exon		296	del1			FS	0	Pro
23165	g.13854a>t	c.874	8-exon	p.K292X	292	A>T	TAA	Stop	nonsense	2	Pro
23167	g.13379c>t	c.742	7-exon	p.R248W	248	C>T	TGG	Trp	missense	654	Arg
23172	g.14066g>t	c.993 + 1	9-intron		0	G>T			splice	12	Arg
23187	g.13798g>a	c.818	8-exon	p.R273H	273	G>A	CAT	His	missense	733	Arg
23197	g.11550t>g	c.319	4-exon	p.Y107D	107	T>G	GAC	Asp	missense	3	Arg/Pro
23204†	g.16915c>t	c.1024	10-exon	p.R342X	342	C>T	TGA	Stop	nonsense	59	Arg/Pro
23213‡											Arg
23221											Arg
26047	g.16915_16924delCGAGAGCTGA	c.1024	10-exon		342	del10			FS	0	Pro
27006	g.12728a>g	c.659	6-exon	p.Y220C	220	A>G	TGT	Cys	missense	312	Pro
27098	g.16915c>t	c.1024	10-exon	p.R342X	342	C>T	TGA	Stop	nonsense	59	Arg
32022	g.13373a>g	c.736	7-exon	p.M246V	246	A>G	GTG	Val	missense	46	Arg
32032†	g.13798g>a	c.818	8-exon	p.R273H	273	G>A	CAT	His	missense	733	Arg
32049	g.13848_13851delCGCA	c.868	8-exon		290	del4			FS	0	Arg
32054	g.13352_13358delAACAGTT	c.715	7-exon		239	del7			FS	1	Arg
32055	g.13794g>a	c.814	8-exon	p.V272M	272	G>A	ATG	Met	missense	97	Pro
32089	g.12728a>g	c.659	6-exon	p.Y220C	220	A>G	TGT	Cys	missense	312	Arg
32098	g.12457g>t	c.469	5-exon	p.V157F	157	G>T	TTC	Phe	missense	174	Arg
32103	g.13379c>g	c.742	7-exon	p.R248G	248	C>G	GGG	Gly	missense	23	Arg
34049†	g.12653t>c	c.584	6-exon	p.I195T	195	T>C	ACC	Thr	missense	84	Arg

34066	g.11326a>g	c.97 − 2	3-intron		0	A>G			splice	1	Arg
34078	g.13797c>t	c.817	8-exon	p.R273C	273	C>T	TGT	Cys	missense	611	Arg
34080	g.13798g>t	c.818	8-exon	p.R273L	273	G>T	CTT	Leu	missense	136	Arg
34085	g.12457g>t	c.469	5-exon	p.V157F	157	G>T	TTC	Phe	missense	174	Arg
34102	g.11517delT	c.286	4-exon		96	del1			FS	0	Arg
34172	g.13800g>t	c.820	8-exon	p.V274F	274	G>T	TTT	Phe	missense	27	Arg
34186	g.12713g>t	c.644	6-exon	p.S215I	215	G>T	ATT	Ile	missense	23	Pro
34801	g.13797c>t	c.817	8-exon	p.R273C	273	C>T	TGT	Cys	missense	611	Arg
41023	g.13383g>t	c.746	7-exon	p.R249M	249	G>T	ATG	Met	missense	62	Pro
41064	g.13318_13319insT	c.681	7-exon		227	ins1			FS	0	Pro
41165	g.11525_11528delTTCC	c.294	4-exon		98	del4			FS	0	Arg
41203	g.13812c>t	c.832	8-exon	p.P278S	278	C>T	TCT	Ser	missense	74	Arg
41285‡	g.13794g>a	c.814	8-exon	p.V272M	272	G>A	ATG	Met	missense	97	Pro
41307†											Arg
41310	g.12427delG	c.439	5-exon		147	del1			FS	8	Arg
41358											Arg
44102	g.12728a>g	c.659	6-exon	p.Y220C	220	A>G	TGT	Cys	missense	312	Arg
44110†	g.13338a>g	c.701	7-exon	p.Y234C	234	A>G	TGC	Cys	missense	123	Pro
44286	g.12524a>g	c.536	5-exon	p.H179R	179	A>G	CGT	Arg	missense	139	Arg
44303	g.13991g>a	c.920 − 1	8-intron		0	G>A			splice	7	Arg
44377	g.13824c>t	c.844	8-exon	p.R282W	282	C>T	TGG	Trp	missense	519	Arg
44428	g.13379c>t	c.742	7-exon	p.R248W	248	C>T	TGG	Trp	missense	654	Arg
51086	g.12653t>a	c.584	6-exon	p.I195N	195	T>A	ACC	Asn	missense	14	Arg
51093	g.12683a>g	c.614	6-exon	p.Y205C	205	A>G	TGT	Cys	missense	106	Pro
51104‡											Arg
51125	g.13350g>a	c.713	7-exon	p.C238Y	238	G>A	TAT	Tyr	missense	79	Arg
51171	g.11596_11597delTG	c.365	4-exon		122	del2			FS	2	Pro
60016	g.12457g>t	c.469	5-exon	p.V157F	157	G>T	TTC	Phe	missense	174	Arg
60020	g.11489_11501delACCAGCCCCCTCC	c.258	4-exon		86	del13			FS	0	Arg
60024	g.12679delG	c.610	6-exon		204	del1			FS	2	Pro
60048	g.12512g>a	c.524	5-exon	p.R175H	175	G>A	CAC	His	missense	1063	Arg
60049											Arg
60066	g.13407t>a	c.770	7-exon	p.L257Q	257	T>A	CAG	Gln	missense	14	Arg
60067	g.12659delT	c.590	6-exon		197	del1			FS	0	Arg
60090	g.12714t>g	c.645	6-exon	p.S215R	215	T>G	AGG	Arg	missense	14	Arg
60102†	g.12367t>c	c.379	5-exon	p.S127P	127	T>C	CCC	Pro	missense	5	Arg
60107†	g.13822a>g	c.842	8-exon	p.D281G	281	A>G	GGC	Gly	missense	15	Pro
60111†											Arg
60163	g.12512g>a	c.524	5-exon	p.R175H	175	G>A	CAC	His	missense	1063	Arg
60169†	g.13804g>a	c.824	8-exon	p.C275Y	275	G>A	TAT	Tyr	missense	70	Pro
60175	g.12727t>c	c.658	6-exon	p.Y220H	220	T>C	CAT	His	missense	14	Arg
60181†	g.13412g>t	c.775	7-exon	p.D259Y	259	G>T	TAC	Tyr	missense	29	Arg
60188	g.12530_12553delGCTGCTCAGATAGCGATGGTGAGC	c.542	5-exon		181	del24			FS	0	Arg
60211	g.13407t>a	c.770	7-exon	p.L257Q	257	T>A	CAG	Gln	missense	14	Arg
60214											Arg
60258											Arg
70005	g.16918g>t	c.1027	10-exon	p.E343X	343	G>T	TAG	Stop	nonsense	5	Arg
70039	g.13370g>a	c.733	7-exon	p.G245S	245	G>A	AGC	Ser	missense	396	Arg
70045	g.13991g>a	c.920 − 1	8-intron		0	G>A			splice	7	Arg
70077	g.12706c>t	c.637	6-exon	p.R213X	213	C>T	TGA	Stop	nonsense	259	Arg

70100	g.13308a>g	c.673 − 2	6-intron		0	A>G			splice	12	Arg
70103	g.16915c>t	c.1024	10-exon	p.R342X	342	C>T	TGA	Stop	nonsense	59	Arg
70107	g.13359c>t	c.722	7-exon	p.S241F	241	C>T	TTC	Phe	missense	94	Pro
70118	g.12722t>g	c.653	6-exon	p.V218G	218	T>G	GGG	Gly	missense	12	Arg
70123	g.16915c>t	c.1024	10-exon	p.R342X	342	C>T	TGA	Stop	nonsense	59	Arg
70125	g.12505g>a	c.517	5-exon	p.V173M	173	G>A	ATG	Met	missense	60	Arg
70135	g.13798g>t	c.818	8-exon	p.R273L	273	G>T	CTT	Leu	missense	136	Pro
