# Supplementary material for: Driver mutations in TP53 are ubiquitous in high grade serous carcinoma of the ovary
Source: J Pathol. 2010 May;221(1):49–56. doi: 10.1002/path.2696 (PMC3262968; doi:10.1002/path.2696)
Supplement: Supplementary file 2 [file path0221-0049-SD2.pdf]

Supplementary Table 2. Summary of clinical, pathology and molecular review of mutation-negative serous carcinomas.

Study ID	Cohort	Original Diagnosis	Pathology Review	Clinical Review	p53 Staining	Genomic Change	*MDM2* Copy Number	*MDM4* Copy Number	*TP53* Copy Number	Tumour Content
533	Pilot	Stage IIIC, grade 2, papillary serous adenocarcinoma	Grade 2 with areas of grade 1 and co-existing borderline tumor	Age 22 yr at diagnosis, poor treatment response	Negative	1p loss				>90%
22029	Validation	Stage IV, grade 3, papillary serous adenocarcinoma	Original report confirmed	Pathogenic germline *BRCA2* mutation	Heterogeneously positive (+ to +++)	Moderately aberrant		Gain		>70%
23221	Validation	Stage IIIC, grade 3, serous carcinoma	High-grade tumour of uncertain primary site (differential diagnosis; serous carcinoma, mesothelioma and gastrointestinal tract carcinoma)	Neoadjuvant chemotherapy, platinum-refractory	Negative (stromal cells occasional +)	Highly aberrant				>60%
41358	Validation	Stage IIIC, grade 3, serous carcinoma	Original report confirmed		90% strongly positive (+++)	Highly aberrant			Deletion	>60%
60049	Validation	Stage IIIC, grade 3, serous carcinoma	Original report confirmed		Heterogeneously focal (+ to +++) and 80% cytoplasmic (+)	Highly aberrant		Gain	Deletion	>60%
60214	Validation	Stage IIIC, grade 2, serous carcinoma	Grade 1 invasive		30% Positive (++)	Aneuploidy		Gain	Deletion	>70%
60258	Validation	Stage IIIC, grade 3, papillary serous adenocarcinoma	Original report confirmed		90% strongly positive (+++)	Highly aberrant	Gain	Deletion	Deletion	>70%
