# Supplementary material for: Driver mutations in TP53 are ubiquitous in high grade serous carcinoma of the ovary
Source: J Pathol. 2010 May;221(1):49–56. doi: 10.1002/path.2696 (PMC3262968; doi:10.1002/path.2696)
Supplement: Supplementary file 3 [file path0221-0049-SD3.pdf]

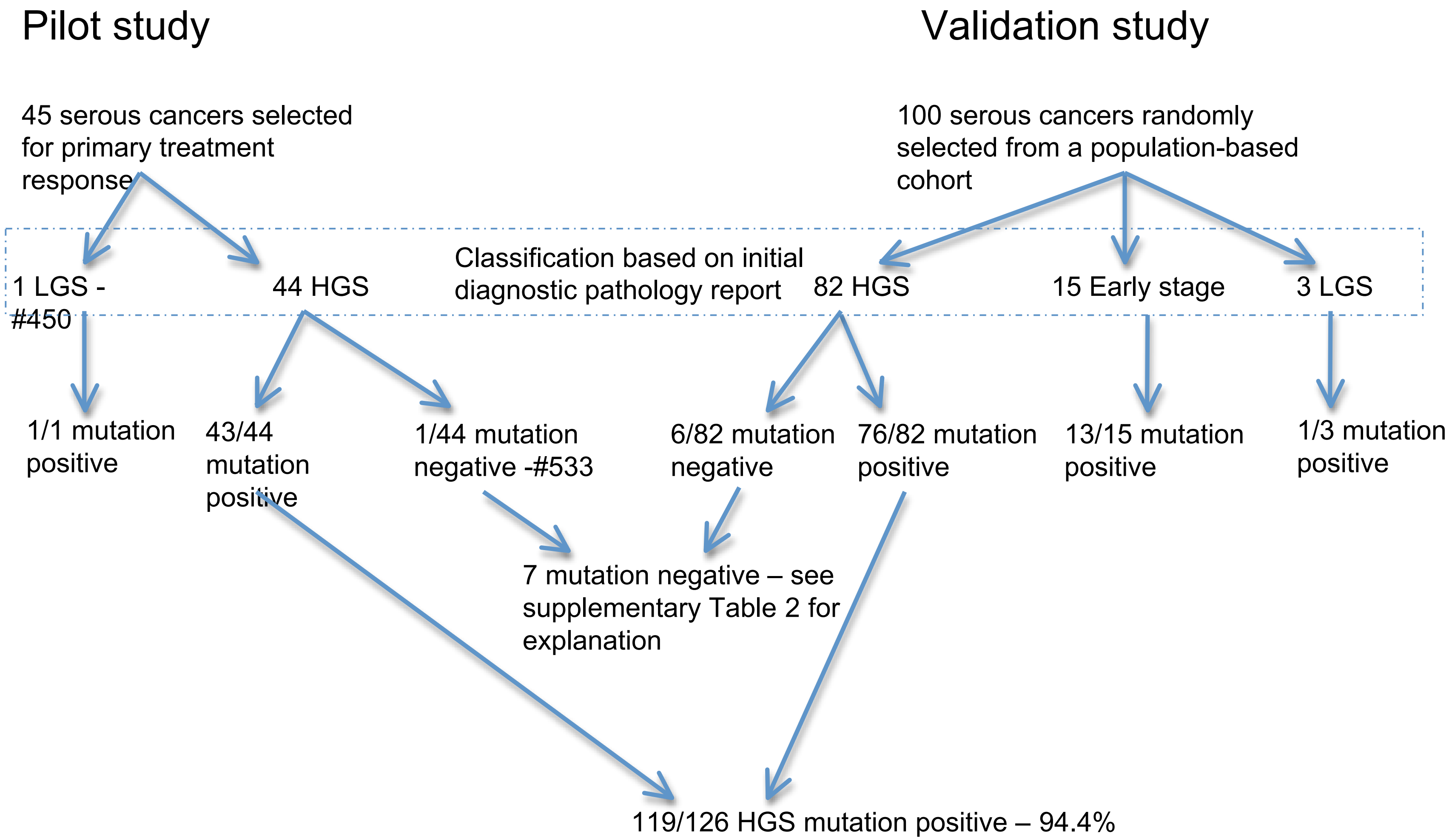

Supplementary Figure 1. Summary of all cases included in the pilot and validation sets. Following the recommendations of the REMARK guidelines we have reported the outcome of analysis of all cases in the study and have then focused on those that fulfilled the criteria of high grade (grade 2-3) late stage (FIGO stage III-IV) serous invasive ovarian cancers.
