# Supplementary material for: Driver mutations in TP53 are ubiquitous in high grade serous carcinoma of the ovary
Source: J Pathol. 2010 May;221(1):49–56. doi: 10.1002/path.2696 (PMC3262968; doi:10.1002/path.2696)
Supplement: Supplementary file 4 [file path0221-0049-SD4.doc]

**SUPPLEMENTARY METHODS**

**Samples and study design**

Our initial aim was to conduct a study of sufficient power to detect a 20% difference in the two-year recurrence rate in patients with or without *TP53* mutations. Previous studies showed that the 2-year recurrence rate in ovarian cancer is approximately 73% [39]. We hypothesized that this rate may represent the mean of the two-year recurrence rate in patients with TP53 mutation (presumed higher recurrence rate of 83%) and the two-year recurrence rate in patients with no TP53 mutations (presumed lower recurrence rate of 63%). We estimated a *TP53* mutation rate of 50% from the approximate median value of published reports. We therefore powered the study to detect the difference in two-year progression-free survival of between 63% and 83%. To detect this difference with 80% power at p<0.05 using a two-sided t test, we estimated that 85 samples were required in each arm. A pilot study was designed to test the assumptions made for our power calculations, based on 45 patients who had primary invasive, high-grade (2 or 3), advanced stage (FIGO stage III or IV) tumours of serous histology. All patients underwent debulking surgery and received platinum-based chemotherapy, the majority in the adjuvant setting (n = 1, received neoadjuvant chemotherapy). Patients were selected according to treatment response as previously described [40] and were categorized as platinum-resistant or treatment-responsive if the progression-free interval from the end of primary treatment was less than six months or greater than nine months, respectively. Progressive disease was determined by CA125 marker or imaging according to RECIST (Response Evaluation Criteria in Solid Tumours) guidelines adapted for ovarian cancer [41,42] or by clinical examination. Progression-free survival was defined as the time interval between the date of histological diagnosis and the first confirmed sign of disease recurrence or progression. Overall survival was calculated from the date of histological diagnosis to the date of death or last contact.

A validation group of invasive serous cancers cases was randomly drawn from the AOCS cohort and were not selected based on treatment response. We assumed that individuals in the study were independent and *a priori* have equal probability of mutation. In order to have 80% power to reject the null hypothesis that the rate of *TP53* mutation in HGS cancer was not significantly higher than 78% [43], we estimated that an additional 37 samples were required. This allowed the detection of the difference 95% vs 78% at *P* < 0.001. To account for possible variations in the reported rate of *TP53* mutations we randomly selected and sequenced a total of 100 cases. Sequencing and analysis was performed blinded to the clinical characteristics of the validation cohort. Haematoxylin and eosin (H&E) stained tissue sections were reviewed by pathologists specialized in gynaecological cancer, to confirm the histological subtype, and cases were re-graded according to standardized criteria [44]. On review of primary data, 82/100 cases fulfilled the prospectively defined criteria of HGS carcinoma with advanced stage (FIGO III or IV) and histological grade of 2 or 3, and were included in the final analysis (Table 2 and Supplementary Figure 1). Comparison of the clinical characteristics of the 82 HGS cases in the validation group with other serous cancers in the AOCS study showed that they were representative of the entire cohort (data not shown). As AOCS is a population-based series, the validation group is representative of serous ovarian cancers derived from an ethnically diverse Western population.

**DNA Sequencing protocols**

The following precautions were taken to ensure the identification of true *TP53* mutations: 1) each mutation was identiﬁed using a forward and a reverse sequencing reaction, 2) each mutation was subsequently conﬁrmed using independent sequencing reactions (forward and reverse directions), 3) twelve of the rare mutations from the pilot study (n = 12) was conﬁrmed by an independent reference laboratory that was blinded to the mutation status, (Cancer Research UK Sequencing Facility, Leeds), 4) seven cases with complex insertions or deletions were cloned into pCR2.2-TOPO (Invitrogen) to confirm correct sequence, 5) all reagents were used pre-aliquoted, only once, with fresh reagents prepared at every new batch of PCR reactions, 6) all PCR reactions were performed in a dedicated laminar ﬂow hood and PCR equipment to minimize the possibility of cross contamination, 7) each of the resulting sequence traces was hand-examined by two members of the research team to confirm high quality sequence reads, the minimum quality criterion for confirming mutations was three high quality electropherograms obtained from two independent amplicons, 8) all putative mutations were compared with the IARC *TP53* mutation database and, 9) eighty-five validated SNP polymorphisms were excluded from the analysis (http://www-p53.iarc.fr/Polymorphism.html).

**Tissue Processing and DNA Extraction**

Tumor cellular composition of tissue collected at primary surgery was assessed by a pathologist using H&E-stained sections that flanked the fresh-frozen tissue used for DNA extraction. Where the neoplastic cell content was greater than 80%, DNA was extracted from whole tumor material. For the remaining cases, serial 16 µm tumor sections were first stained with cresyl-violet (Ambion) and needle-dissected to enrich for the epithelial fraction. Genomic DNA was extracted using a DNeasy kit (Qiagen) according to the manufacturer’s protocol. DNA was quantified and quality assessed by spectrophotometry.

***TP53* Sequencing and mutation detection – pilot cohort**

*TP53* exons 2 to 11 were amplified using primers covering the exons and exon-intron boundaries (Table 1). PCR was performed in 50 μl reactions containing 1.5 mM MgCl_2_, 200 μM dNTPs, 0.2 μM each primer, 1.5 units AmpliTaq Gold (Applied Biosystems) and 10 ng DNA. PCR cycling conditions were as follows: 1 cycle of 95°C for 10 min; 35 cycles of 95°C for 30 sec, 55-60°C (exon dependent) for 30 secs, 72°C for 30 sec; 72°C for 10 min. PCR reactions were cycled in a DNA Engine Tetrad, PTC-225 thermal cycler (Biorad, Hercules, CA). PCR products were purified using the NucleoFast 96 PCR plates from Macherey-Nagel using the manufacturer's protocol. Sequencing reactions were carried out using BigDye terminator kit v.3.1 (Applied Biosystems), dye terminators were removed using isopropanol precipitation and sequence reactions resolved on ABI PRISM® 3100 Genetic Analyzer.

Following sequencing of the pilot set, sequencing conditions were slightly modified to enhance sequence output, reduce cost and improve efficiency.

***TP53* Sequencing and mutation detection – Validation cohort**

*TP53* exons 2 to 11 were amplified using primers covering the exons and exon-intron boundaries (Table 1). PCR was performed in 50 μl reactions containing 1.5 mM MgCl_2_, 200 μM dNTPs, 0.3 μM each primer, 0.5 units Expand High Fidelity (Roche). PCR cycling conditions were as follows: 1 cycle of 94°C for 2 min; 10 cycles of 94°C for 15 sec, 55-67°C (exon dependent) for 30 sec, 72°C for 45 sec; 20 cycles of 94°C for 15 sec, 55-67°C (exon dependent) for 30 sec, 72°C for 45 sec (plus 5 sec for each successive cycle); 72°C for 7 min. PCR reactions were cycled in a DNA Engine Tetrad 2, Peltier thermal cycler (Biorad). PCR products were diluted before sequencing reactions were carried out using BigDye terminator kit v.3.1 (Applied Biosystems), dye terminators were removed using Performa DTR V3 96-well short plate kit (EdgeBio) and sequence reactions resolved on ABI PRISM® 3130xl Genetic Analyzer.

***KRAS*, *BRAF*, *CTNNB1* and *PIK3CA*.**

The methods and primers used for sequencing were previously described [45,46].

**Single-Nucleotide Polymorphism (SNP) Mapping Assay**

Affymetrix 6.0 SNP mapping assays were performed according to the manufacturer’s ‘Cytogenetics Copy Number Assay’ protocol (Affymetrix). Genomic DNA was digested with *StyI* or *NspI* endonucleases, adapter ligated and PCR amplified. Pooled PCR products were then purified, fragmented with DNaseI, biotin labelled, denatured and hybridized to Affymetrix 6.0 SNP Mapping Arrays. The arrays were then washed on the Affymetrix fluidics station 450, stained with streptavidin-phycoerythrin and scanned using a GeneChip® Scanner 3000.

**SNP Microarray Data Analysis**

Data files (CEL) were generated using the Affymetrix GeneChip® Command Console (AGCC) v1.0 and normalized using the R package Aroma. Affymetrix [47]. Allelic cross talk calibration and fragment length normalization steps were implemented to improve the signal to noise ratio. DNA copy number at each marker was then estimated by normalization to the mean signal obtained from normal reference samples profiled at the Peter MacCallum Cancer Centre. Data was visualized using Partek Genomics Suite 6.4.

**Quantitative-PCR (qPCR)**

*MDM2* and *MDM4* gene copy number was measured by SYBR green qPCR using the 7900HT Fast Real-Time PCR system (Applied Biosystems). Each gene was measured in triplicate 10 μL reactions containing 2 ng of genomic DNA, 1 μM of each forward and reverse primer, and SYBR green master mix (Applied Biosystems). Amplification conditions were; 50°C for 2 min, 95°C for 10 min followed by 40 cycles of 95°C for 15 sec and 60°C for 1 min. Two sets of primer pairs were designed per gene using Primer3 [48] or obtained from previous studies [49] as shown in Supplementary Methods. Disassociation analysis was performed in Sequence Detection Software v 2.3 (Applied Biosystems) to ensure amplification product specificity. Target gene quantity is given as the average log_2_ ratio value obtained for each primer pair after normalization of threshold cycle values to the repetitive element Line-1 and a normal DNA reference [50].

**Immunohistochemistry**

Immunohistochemical staining was performed according to standard procedures with Envision Flex kit reagents (Dako) and a p53 monoclonal antibody (clone DO-7, Novocastra). Slides were first de-waxed and re-hydrated followed by heat antigen retrieval (100°C for 20 min). Blocking of endogenous peroxidases, staining and wash steps were performed in a Dako autostainer. Primary antibody incubation was for 30 min at a 1/400 dilution. HRP detection reagent and DAB+ chromogen were applied followed by counterstaining with Mayer’s Haematoxylin. After staining, slides were dehydrated, cleared and mounted in mounting medium (Pertex). Staining was assessed by a pathologist and images taken using an Olympus BX51 microscope.

**References**

**(Note: reference numbers correspond to reference list in main article)**

1. ICON. Paclitaxel plus carboplatin versus standard chemotherapy with either single-agent carboplatin or cyclophosphamide, doxorubicin, and cisplatin in women with ovarian cancer: the ICON3 randomised trial. *Lancet* 2002;360:505-515.
2. Etemadmoghadam D, deFazio A, Beroukhim R, Mermel C, George J, Getz G, *et al.* Integrated genome-wide DNA copy number and expression analysis identifies distinct mechanisms of primary chemoresistance in ovarian carcinomas. *Clin Cancer Res* 2009;15:1417-1427.
3. Rustin GJ, Nelstrop AE, McClean P, Brady MF, McGuire WP, Hoskins WJ, *et al.* Defining response of ovarian carcinoma to initial chemotherapy according to serum CA 125. *J Clin Oncol* 1996;14:1545-1551.
4. Vergote I, Rustin GJ, Eisenhauer EA, Kristensen GB, Pujade-Lauraine E, Parmar MK, *et al.* Re: new guidelines to evaluate the response to treatment in solid tumors [ovarian cancer]. Gynecologic Cancer Intergroup. *J Natl Cancer Inst* 2000;92:1534-1535.
5. Kupryjanczyk J, Thor AD, Beauchamp R, Merritt V, Edgerton SM, Bell DA, *et al.* p53 gene mutations and protein accumulation in human ovarian cancer. *Proc Natl Acad Sci U S A* 1993;90:4961-4965.
6. Shimizu Y, Kamoi S, Amada S, Akiyama F, Silverberg SG. Toward the development of a universal grading system for ovarian epithelial carcinoma: testing of a proposed system in a series of 461 patients with uniform treatment and follow-up. *Cancer* 1998;82:893-901.
7. Parsons DW, Jones S, Zhang X, Lin JC, Leary RJ, Angenendt P, *et al.* An integrated genomic analysis of human glioblastoma multiforme. *Science* 2008;321:1807-1812.
8. Kuo KT, Mao TL, Jones S, Veras E, Ayhan A, Wang TL, *et al.* Frequent activating mutations of PIK3CA in ovarian clear cell carcinoma. *Am J Pathol* 2009;174:1597-1601.
9. Bengtsson H, Irizarry R, Carvalho B, Speed TP. Estimation and assessment of raw copy numbers at the single locus level. *Bioinformatics* 2008;24:759-767.
10. Rozen S, Skaletsky H. Primer3 on the WWW for general users and for biologist programmers. *Methods Mol Biol* 2000;132:365-386.
11. Riemenschneider MJ, Buschges R, Wolter M, Reifenberger J, Bostrom J, Kraus JA, *et al.* Amplification and overexpression of the MDM4 (MDMX) gene from 1q32 in a subset of malignant gliomas without TP53 mutation or MDM2 amplification. *Cancer Res* 1999;59:6091-6096.
12. Wang TL, Maierhofer C, Speicher MR, Lengauer C, Vogelstein B, Kinzler KW, *et al.* Digital karyotyping. *Proc Natl Acad Sci U S A* 2002;99:16156-16161.

**Table 1: Primers used for *TP53* sequencing:**

| **Exon** | **Primer Sequence (forward)** | **Primer Sequence (reverse)** | **Tm (ºC)** | **Product Size (bp)** |
| --- | --- | --- | --- | --- |
| 2 | cgagctgtctcagacactgg | ccttgtccttaccagaacgttg | 58 | 366 |
| 3 | cat ggg act gac ttt ctg ctc ttg | cgg gga cag cat caa atc atc | 55 | 208 |
| 4 | ctg gtc ctc tga ctg ctc tt | cat tga agt ctc atg gaa gc | 58 | 355 |
| 5 | ctc tgt ctc ctt cct ctt cc | gca atc agt gag gaa tca gag g | 55 | 284 |
| 6 | aga tag cga tgg tga gca gc | act gac aac cac cct taa cc | 60 | 258 |
| 7 | cag gtc tcc cca agg cgc ac | gca agc aga ggc tgg ggc ac | 60 | 219 |
| 8 | gga gta gat gga gcc tgg tt | gtg aat ctg agg cat aac tg | 58 | 287 |
| 9 | ggg tgc agt tat gcc tca gat t | cgg cat ttt gag tgt tag act gg | 55 | 190 |
| 10 | ctt ctc ccc ctc ctc tgt tgc | gaa ggc agg atg aga atg ga | 58 | 218 |
| 11 | tgg tca ggg aaa agg ggc ac | gag aga tgg ggg tgg gag gc | 58 | 205 |

**Table 2: Primers used for quantitative PCR of *MDM2* and *MDM4*:**

| **Gene** | **Primer Set** | **Primer Sequence (forward)** | **Tm (ºC)** | **Primer Sequence (reverse)** | **Tm (ºC)** | **Product Size (bp)** |
| --- | --- | --- | --- | --- | --- | --- |
| *MDM2* | 1 | cggaaagatggagcaagaag | 60.0 | gcgctcgtacgcactaatc | 59.6 | 116 |
| *MDM2* | 2 | gacaaagaaaacgccacaaa | 58.8 | atggcgtccctgtagattca | 60.5 | 123 |
| *MDM4 [50]* | 1 | gagtggcagtgtactgaatgc | 58.4 | gcatctttaggtctaacgacag | 55.9 | 221 |
| *MDM4* | 2 | tggaccttggatttgaggag | 60.0 | tgtctgtaaatcagttgagccatt | 60.1 | 110 |
| Line-1^2^ | 1 | aaagccgctcaactacatgg | 60.3 | tgctttgaatgcgtcccagag | 65.8 | 149 |
